# ID1 As a Prognostic Biomarker and Promising Drug Target Plays a Pivotal Role in Deterioration of Clear Cell Renal Cell Carcinoma

**DOI:** 10.1155/2020/2064582

**Published:** 2020-10-29

**Authors:** Xiangmin Qiu, Yuping Gu, Yilu Ni, Qianyin Li

**Affiliations:** The Ministry of Education Key Laboratory of Laboratory Medical Diagnostics, The College of Laboratory Medicine, Chongqing Medical University, 400016 Chongqing, China

## Abstract

Clear cell renal cell carcinoma (ccRCC) is one of the most common cancers in the world. Our aim is to identify prognostic biomarkers that contribute to the progression of early stage ccRCC and clarify the mechanism. Here, the mRNA microarray expression profile of ccRCC samples was obtained from Gene Expression Omnibus (GEO) (GSE68417). 62 differentially expressed genes (DEGs) were gained by R Studio, including 31 upregulated genes and 31 downregulated genes. Pathway enrichment analysis was performed in DAVID database. Then, the protein-protein interaction network was obtained through STRING database and visualized by Cytoscape. Subsequently, among the network, only inhibitor of DNA Binding 1 (ID1) was significant between low-grade and high-grade ccRCC patients in TCGA data set. After analysis of the corresponding clinical information in R Studio, it is shown that low ID1 expression correlated with poor survival, high probability of tumor metastasis, and relatively high serum calcium. Later, functional enrichment of ID1 in GeneMANIA uncovered that regulating DNA binding is a main characteristic of ID1 in ccRCC, which was validated by Kaplan-Meier curve of ID1 associated genes using KM plotter database and R Studio. Immune infiltration analysis performed by Tumor Immune Estimation Resource (TIMER) revealed that CD8+ T cells and macrophages were prognostic factors. Furthermore, Valproic acid was analyzed to be the most convinced target drug of ID1 identified by Comparative Toxicogenomics Database (CTD). Taken together, ID1, a biomarker of clinical outcome in early stage ccRCC patients, has the potential function of preventing deterioration in ccRCC progression and metastasis.

## 1. Introduction

Renal cell carcinoma (RCC) ranks among the top ten of the most commonly diagnosed cancers worldwide [1]. In all cases, clear cell renal cell carcinoma (ccRCC) covers 75% and is the majority of cancer-associated deaths, proving it to be major histology of RCC [2]. Some angiogenesis inhibitors are often characterized by off-target effects and chronic toxicities such as fatigue and rash. Furthermore, the metabolic basis of ccRCC is only partially addressed by targeting the terminal phenotype driven by VEGF [3]. Moreover, recent studies mainly focus on tumor initiation but pay little attention to tumor deterioration. Thus, searching for biomarkers associated with tumor deterioration and clarifying the mechanism are of great importance.

Presently, some public databases like The Cancer Genome Atlas (TCGA) and Gene Expression Omnibus (GEO), which contain microarrays, RNAseqs and clinical information of diseases, are comprehensive resources. Bioinformatics tools such as DAVID, STRING, GeneMANIA, and TIMER are useful tools to perform enrichment of genes, protein-protein interaction, and immune infiltration. With the aid of bioinformatic analysis, some prognostic biomarkers are reported, such as AHR, GRHL2, KIAA0101, AQP1, DDX11, and BAIAP2L1 [4–7]. However, the key biomarkers in ccRCC progress and the mechanism of dissemination are little reported. Our aim is to identify novel biomarkers to clarify the disease development and metastasis.

In this study, we analyzed genes expression profiles from Gene Expression Omnibus (GEO) and gained differentially expressed genes (DEGs) of samples of low-grade and high-grade ccRCC. Subsequently, GO and KEGG analyses were performed to determine the functions of these genes. Based on the potential biomarkers, we analyze each of them and identify the inhibitor of DNA binding 1 (ID1) as the survival-associated gene. ID1 gene encodes protein helix-loop-helix (HLH), and this ID1 protein forms heterodimers with members of the basic HLH family of transcription factors. ID1 protein has no DNA binding activity and therefore can inhibit the DNA binding and transcriptional activation ability of basic HLH proteins interacted with ID1 [8]. It is reported that ID1 is associated with tumorigenesis and tumor metastasis [9], but how ID1 affects ccRCC still remains unexplained. Later, function enrichment, clinical indicators analysis, immune infiltration, and target drugs were further performed to shed light on the potential function of ID1.

## 2. Materials and Methods

### 2.1. Data Set Download and Data Preprocessing

The gene expression profile of GSE68417 (Bryan Thibodeau et al. 2015) was collected from GEO database (https://www.ncbi.nlm.nih.gov/geo/), the platform is GPL6244. It is a 73 transcriptional profiling of a clear cell renal cell carcinoma base 49 patient samples associated with different tumor grades. We chose low-grade (Fuhrman grades 1 and 2) and high-grade (Fuhrman grades 3 and 4) samples from GSE68417 as our raw data. Then, the “ID_REF” was transformed into gene symbol. The TCGA data associated with 537 patients at different stages (https://cancergenome.nih.gov/) was downloaded for further gene filtration and clinical manifestation analysis. The UCSC Cancer Genomics Browser (http://xena.ucsc.edu/) [10, 11] was used to download the expression profile of ID1 associated genes.

### 2.2. Screening DEGs of ccRCC

The limma package in R Studio (version 1.2.1335) was used to screen DEGs between low-grade and high-grade groups [12]. The DEGs of the data with an absolute Log2 fold change (FC) > 1 and a *P* value < 0.05 was considered as a cut-off criteria (all DEGs are corrected with FDR < 0.05).

### 2.3. Gene Ontology (GO) Analysis and Kyoto Encyclopedia of Genes and Genomes (KEGG) Analysis

Gene Ontology (GO) analysis is a useful way to unify the biology of genes [13], which can be divided into three categories based on the extracted coexpressed genes: molecular functions (MF), biological processes (BP), and cellular components (CC). We performed GO analysis at functional level by ClusterProfiler package in R Studio [14]. The Kyoto Encyclopedia of Genes and Genomes (KEGG) analysis accomplished by the DAVID database (https://david.ncifcrf.gov/) [15] systematically studies gene functions and combines genomic information with functional information. The cut-off of Benjamini *P* value < 0.05.

### 2.4. Correlation Analysis of ID1 and Clinicopathological Features

The number of patients with lowly or highly expressed ID1 at different stages of clinicopathological characteristics was screened, and the differences were evaluated by the student *t*-test and Kruskal test. *P* values < 0.05 were considered statistically significant.

### 2.5. Establishment of PPI Network

In our study, the STRING database (http://string-db.org) [16] was employed to construct protein-protein interaction network with combined score > 0.4. Then, the network data was imported into the Cytoscape (version 3.7.1) [17] for visualization. The degree of DEGs in network was computed and visualized by ggplot2 package in R Studio (version 1.2.1335). The ID1 functional network was constructed by GeneMANIA [18], which includes annotation for functions and networks.

### 2.6. Overall Survival Analysis

Kaplan-Meier analysis was used to create survival curves. The expression level of ID1 and CXCL8 was determined by survminer package in R. The survival curves of highly or lowly expressed ID1 and CXCL8 groups were analyzed with survival, survminer package in R (version 1.2.1335). The prognostic value of ID1 related genes was analyzed by Kaplan-Meier plotter (http://kmplot.com/analysis/).

### 2.7. Correlation Analysis of ID1 and Immune Cell Infiltration

The infiltration level of immune cells in ccRCC was predicted using the TIMER database (https://cistrome.shinyapps.io/timer/) [19] to estimate the abundance of immune infiltration. The correlation between ID1 expression and the infiltration level of immune cells, including CD8+ T cells, CD4+ T cells, B cells, macrophages, neutrophils, and dendritic cells was analyzed using the Spearman correlation test. Further study was carried out on the correlation between immune cells and ccRCC prognosis in immune infiltration.

### 2.8. Target Drugs for ID1

Comparative Toxicogenomics Database [20] was used to analyze ID1-targeted drugs with reference count > 1, including positive regulated drugs and negative regulated drugs. The selected drugs were visualized by Cytoscape (version 3.7.1), and the bubble diagram of reference count of drugs was plotted by ggplot2 package in R Studio (version 1.2.1335).

## 3. Results

### 3.1. Identification of DEGs in ccRCC

**Figure 1:**
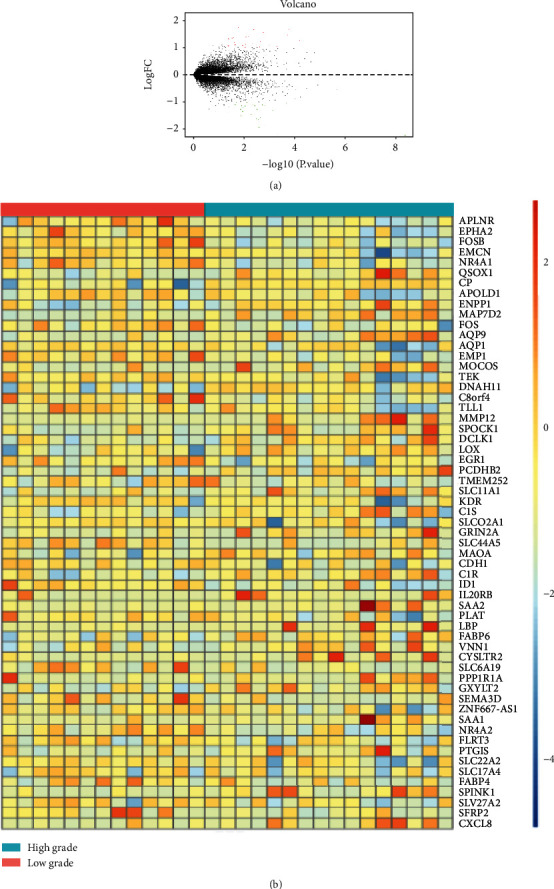
Volcano map, heatmap, and Go analysis of DEGs. (a) Volcano map of DEGs. (b) Heatmap of the DEGs; in each subfigure, red represents upregulated DEGs, and blue represents downregulated DEGs.

We downloaded mRNA microarray data set of GSE68417 from GEO database. 23307 genes were obtained from 49 ccRCC patients including 14 normal samples, 6 benign samples, 13 low-grade samples (Fuhrman grades 1 and 2), and 16 high-grade samples (Fuhrman grades 3 and 4). Then, we selected low-grade samples and high-grade samples from the data set. After that, we used limma packages in R Studio to identify 62 differential genes (absolute Log2 fold change > 1, *P* < 0.05) between higher grade ccRCC and lower grade ccRCC, including 31 upregulated genes and 31 downregulated genes (Figure 1(a)). These DEGs might worsen the symptoms of patients and finally curtail survival time. Next, we used heatmap to visualize the potential biomarkers (Figure 1(b)).

### 3.2. GO Term and KEGG Analysis of DEGs

**Figure 2:**
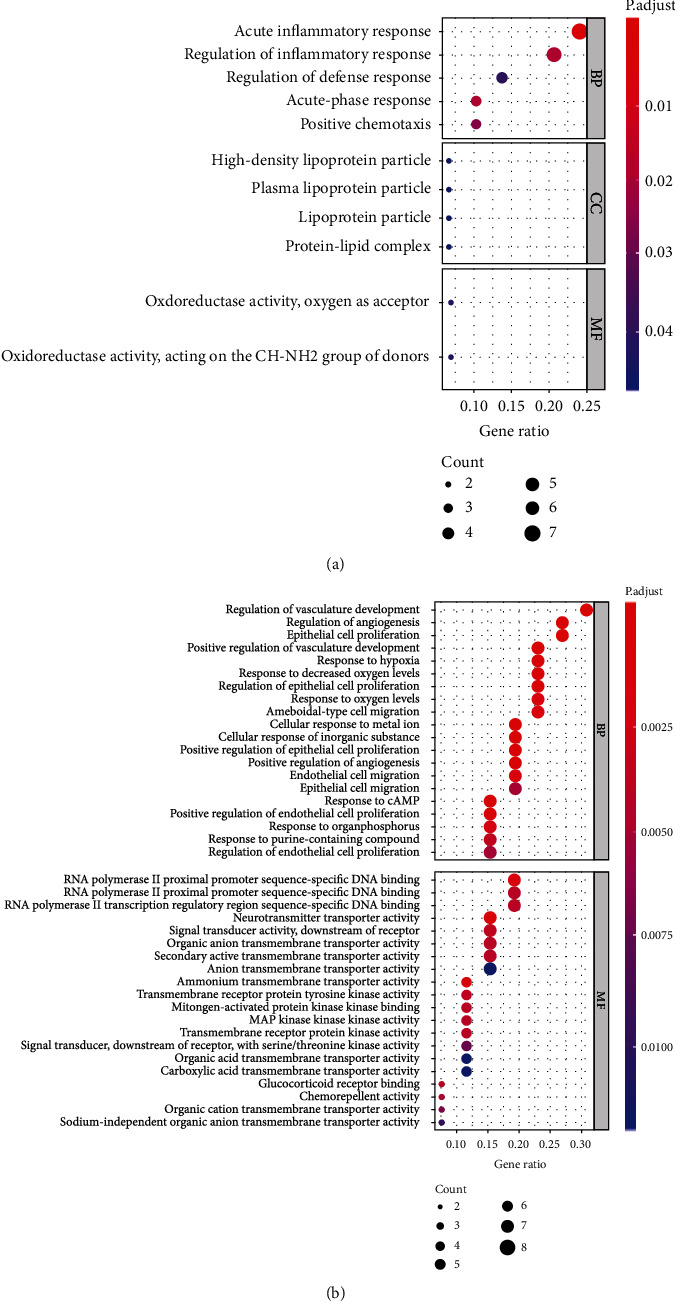
GO analysis of DEGs to enrich genes in related pathways. (a) GO analysis of downregulated DEGs in the DAVID. (b) GO analysis of upregulated DEGs in the DAVID.

To characterize common function of these DEGs, we use Clusterprofiler R packages to perform GO and KEGG analysis. *P* < 0.05 was considered statistically significant. GO categories exhibited that BP (biological process) of downregulated candidates mostly involved in acute inflammatory response (Figure 2(a)). Upregulated DEGs mainly enriched in regulation of vasculature development, regulation of angiogenesis, and epithelial cell development. MF (molecular function) of these genes mainly encompassed DNA binding (Figure 2(b)). According to KEGG analysis, upregulated biomarkers saw significant enrichment in rap1 signaling pathway and MAPK signaling pathway (Table 1). Downregulated genes mostly exhibited enrichment in pertussis and system lupus erythematosus (Table 2).

### 3.3. Discovery of Critical Gene in patient's Survival

**Figure 3:**
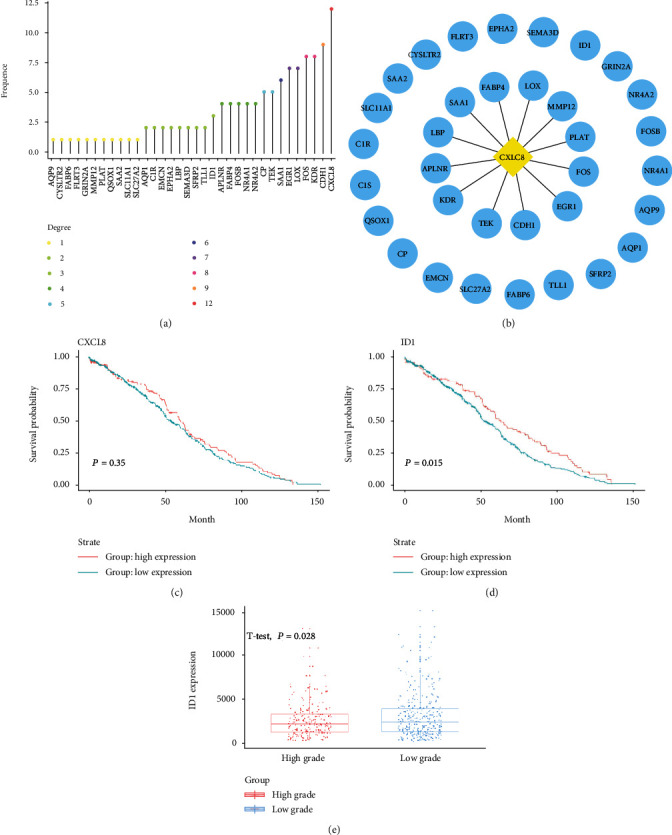
Discovery of critical genes in patient's survival. (a) Distribution of genes and degrees (number of adjacent genes). (b) Genes among DEGs interacted with CXCL8 studied by STRING and visualized by cytoscape-v3.7.1. (c, d) Survival plot of CXCL8 with cut-off value = 0.062 and ID1 with cut-off value = 1.079. Red lines represent high expression of indicated gene, and blue lines represent low expression of indicated gene. (e) Boxplot of ID1 between high-grade and low-grade ccRCC.

To further explore vital genes related to survival of patients, we used STRING to find interaction between DEGs; then, we visualized the interaction of genes in the Cytoscape-v3.7.1 software and computed degree of each DEGs (Figures 3(a) and 3(b)). It exhibited that CXCL8 had the highest degree, which indicated a vital role of CXCL8 in the network. To figure out the survival state of patients between low and high expression of CXCL8, we downloaded data of 537 patients with ccRCC from TCGA (the data includes transcriptome information and clinical information). Later, we divided the CXCL8 expression of patients into high and low levels with a standardized cut-off value (0.062) by using survminer package in R Studio and visualized them with a box plot. The survival curve of patients who suffered from ccRCC was depicted in Figure 2(c). However, we found that there was no significant differentiation of survival time between high-level expression of CXCL8 and the counterpart (*P* = 0.36).

To discover survival-related DEG, survival analysis was applied to all DEGs in Figure 2(a) in the same way as CXCL8-associated survival curve. Luckily, when patients were divided to highly or lowly expressed ID1 groups at a standardized cut-off value (1.079) and the overall survival time between two groups was studied (Figure 3(d)), the data indicated that lowly expressed ID1 was associated with poor overall survival in clear cell renal cell carcinoma (*P* = 0.015). Subsequently, for further clarifying the influence of ID1 on ccRCC progress, 537 ccRCC patients in TCGA database were divided into two grade (327 patients at stage I and stage II as “low grade,” 210 patients at stage III and stage IV as “high grade”), and DEGs were compared between low-grade patients and high-grade patients. Next, student's *t*-test was used to calculate *P* value of these genes. Among them, only ID1 was a significant gene (*P* = 0.028). To determine the alteration of ID1 in ccRCC patients, comparison of ID1 expression was realized using TCGA database between high-grade and low-grade patients. It showed that high-grade patients show lower ID1 expression (Figure 3(e)).

**Figure 4:**
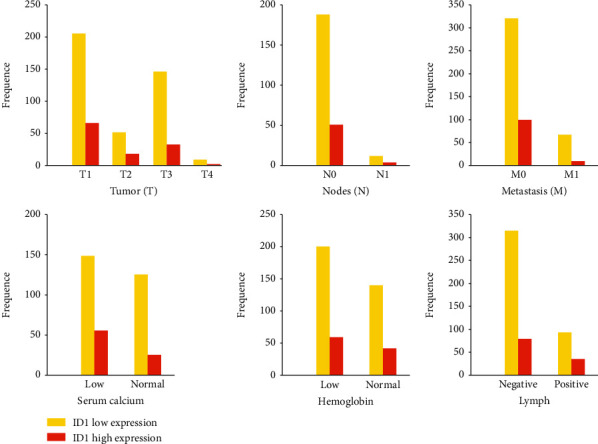
Clinical manifestation of ccRCC patients with lowly- and highly expressed ID1 (cut-off value = 1.079).

After that, other indicators such as Tumor-Node-Metastasis classification (TNM), lymph nodes, serum calcium, and hemoglobin were studied to evaluate the clinical manifestations between lowly expressed ID1 group and highly expressed ID1 group (Figure 4). The results displayed that lowly expressed ID1 was associated with high serum calcium and high probability of metastasis (Table 3).

### 3.4. DNA Binding Regulation of ID1 Plays a Pivotal Role in Deterioration of ccRCC

**Figure 5:**
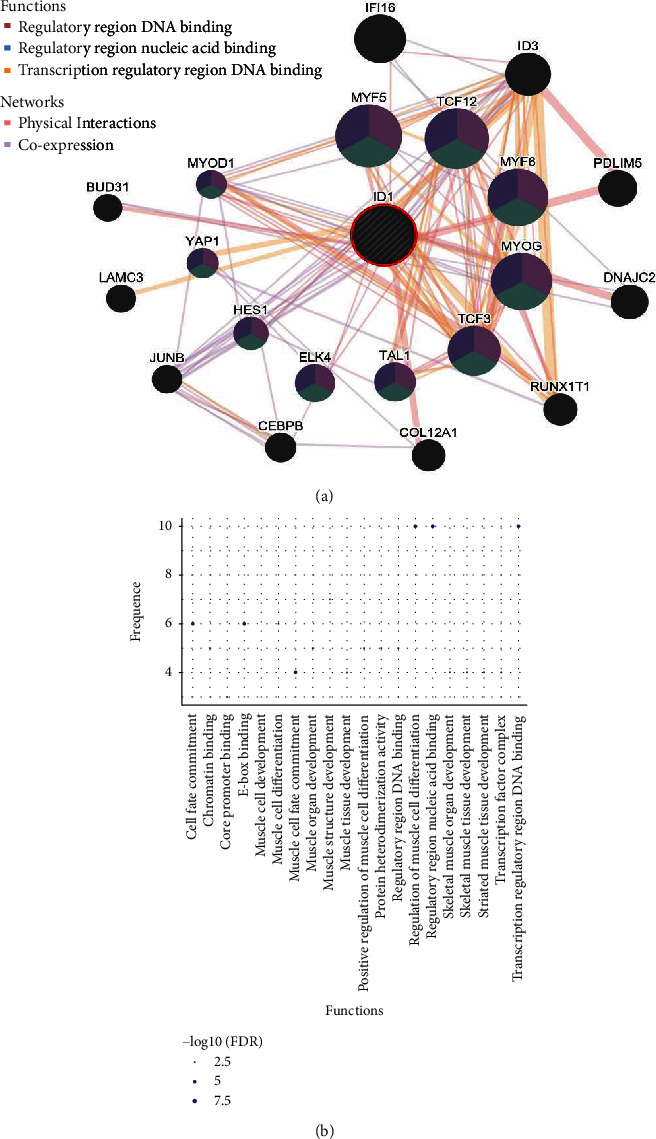
Functional enrichment and KEGG analysis of ID1 associated genes. (a) Genes physically interacted or coexpressed with ID1 was visualized by GeneMANIA; genes in the inner loop have common function: binding to regulatory region nucleic, DNA or transcription regulatory region DNA; genes in the outer loop do not have these functions. (b) KEGG analysis of ID1 associated genes.

**Figure 6:**
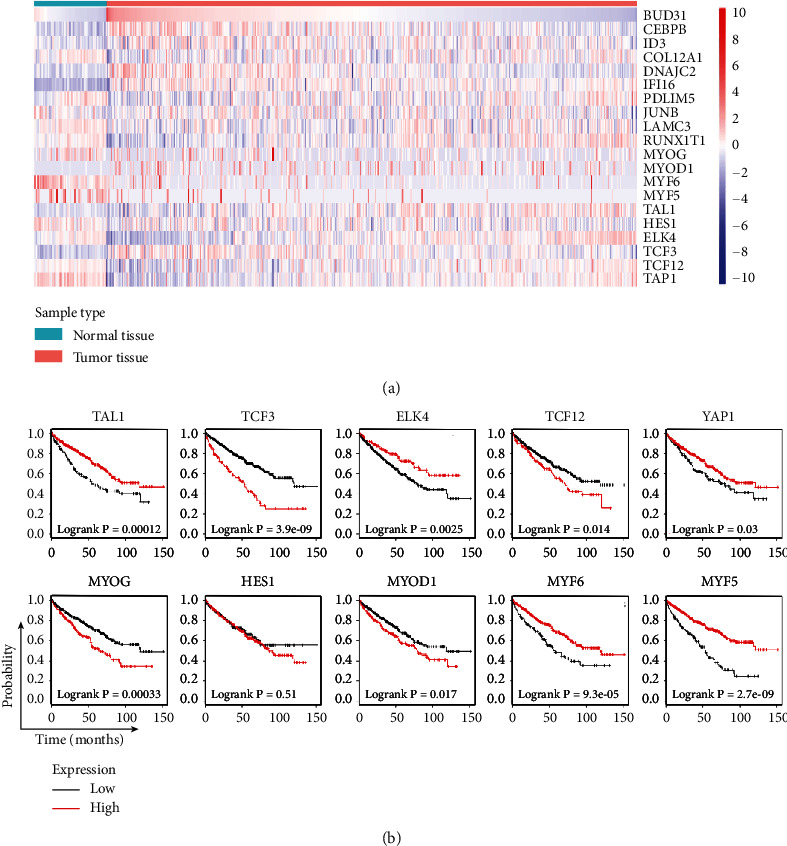
Heatmap and K-M plot of ID1 associated genes indicated ID1-neiborghed genes were associated with patients survival. (a) Heatmap of ID1 associated genes using data from UCSC Xena; red represents upregulated genes, and blue represents downregulated genes. (b) K-M plot of ID1 associated genes.

To find genes which might have synergy with ID1 in procession of ccRCC, ID1's top neighbors were identified using GeneMANIA (Figure 5(a)). Genes in the network have either physical interaction with others or coexpression with others. It was predicted that ID1 and part of ID1-associated genes have common functions in transcription regulation, such as binding to regulatory region nucleic, DNA or transcription regulatory region DNA. The functional enrichment was visualized by bubble diagram (Figure 5(b)). Subsequently, the gene list of ID1-associated genes was gained from UCSC Xena, and the heatmap of these genes were plotted to show the expression difference (Figure 6(a)). Next, the prognostic value of these genes between normal and tumor tissues was analyzed using the Kaplan-Meier plotter (Figure 6(b)). It showed that genes which were associated with ID1 as well as hold DNA binding functions almost have significant prognostic value (except HES1). A recent study confirmed our results that Melatonin inhibits renal cell carcinoma through reduced activity of p65- and p52-DNA-binding [21]. Although survival analyses of ID1-associated genes performed between low-grade patients and high-grade patients were not significant (data not shown), the above outcomes further confirmed that DNA binding regulation is an obvious characteristic of ID1 on alleviating process of ccRCC.

### 3.5. ID1 Related to Patients' Immune System

**Figure 7:**
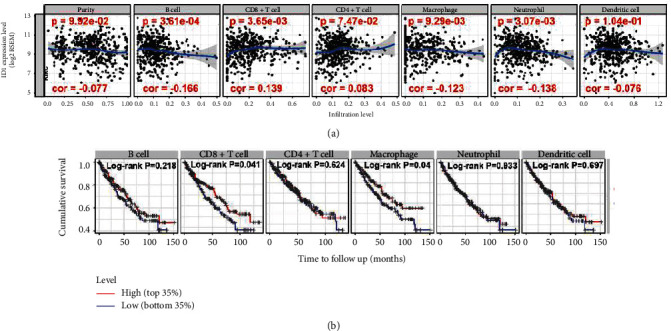
Correlation between ID1 expression and immune infiltration and K-M plot of immune cells using data from TIMER. (a) Correlation between ID1 expression and immune infiltration in the TCGA cohort. (b) Kaplan-Meier survival analysis of immune cells.

Based on the outcome that lowly expressed ID1 might promote cancer cell metastasis and thereby influence patient's survival time, we supposed that ID1 probably affect immune system. Immune infiltration level of immune cells are main parts of immune system, their association with ID1 expression were studied by TIMER. Multivariate Cox analysis showed that CD8 T cell and macrophage cell were independent prognostic value (Table 4). Pearson correlation analysis reviewed a significant positive correlation between ID1 and CD8+ T cells (*r* = 0.139, *P* = 3.65*e* − 3), but not CD4+T cells (*r* = 0.083, *P* > 0.05), and negative correlation between ID1 and B cells (*r* = −0.166, *P* = 3.61*e* − 4), macrophages (*r* = −0.123, *P* = 9.29*e* − 3), and neutrophils (*r* = −0.138, *P* = 3.07*e* − 3), but not tumor homogeneity (*r* = −0.077, *P* > 0.05) and dendritic cells (*r* = −0.078, *P* > 0.05) (Figure 7(a)). The Log-rank test, Kaplan-Meier survival analysis, and multivariate Cox analysis of immune cells infiltration showed that low levels of CD8+ cells and macrophages were significantly associated with poor survival (*P* < 0.05), while B cells, CD4+ T cells, neutrophils, and dendritic cells were not (*P* > 0.05) (Figure 7(b)). These results prompted that ID1-regulating ccRCC progression was associated with tumor immune.

### 3.6. Valproic Acid Might Be Effective to Prevent Deterioration of ccRCC by Increasing ID1 Expression

**Figure 8:**
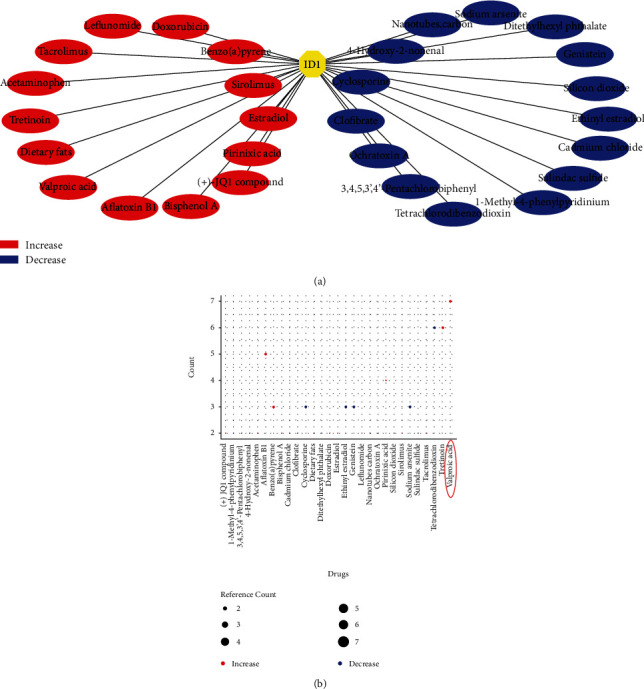
Drug targets of ID1. (a) Analyze drug targets of ID1 using the data from CTD and visualized by cytoscape-v3.7.1; red: drug can increase ID1 expression; blue: drug can decrease ID1 expression; green: the effect of drug was unclear. (b) Reference count of indicated drugs.

To further explore possible drug target of ID1, CTD (Comparative Toxicogenomics Database) was used. The result visualized by Cytoscape reviewed a series of drugs that can target ID1 (Figure 8(a)). The reference count of selected drugs was plotted by R package ggplot2, which revealed that Valproic acid has the highest number of reference count in increasing ID1 expression (Figure 8(b)). Taking it into consideration that ID expression in high-grade ccRCC was lower than in low-grade, we speculated that Valproic Acid probably can prevent ccRCC deterioration and even improve patients' survival status.

## 4. Discussion

ccRCC is a common group of chemotherapy-resistant diseases and can be distinguished by histopathological features and underlying gene mutations. If patients detected early and tumor still localized within the kidney, surgical resection is performed with a 5-year survival rate of ~92% [22]. However, once the tumor has spread locally or systemically, 5-year survival rates drop to 67% or 12%, respectively, as a result of limitations in current therapeutic strategies for metastatic disease [23]. Thus, deciphering the mechanism of disease progression of low-grade patients and metastasis is of great importance.

Here, GEO database was used to download microarray raw data GSE68417 and significant genes were screened, including 31 upregulated genes and 31 downregulated genes. These DEGs might be associated with clinical features and patients' survival (Figure 1). GO and KEGG analysis were employed to define their biological role, which turned out that upregulated DEGs are mainly enriched in the regulation of vasculature development, regulation of angiogenesis, epithelial cell development, DNA binding, rap1 signaling pathway, and MAPK signaling pathway, while downregulated DEGs were mainly involved in acute inflammatory response, pertussis and system lupus erythematosus (Figure 2). It has already been reported that the formation of blood vessels is considered an important mark in tumor and three major types of vessels in ccRCC tumor have been discovered, which is consistent with our results [24]. Next, we performed interaction analysis by using STRING database, and hub gene CXCL8 was selected using Cytoscape (Figures 3(a) and 3(b)). Ha reported that CXCL8-CXCR1/2 axis might make a difference in tumor progression and metastasis by regulating cancer stem cell (CSC) proliferation and self-renewal [25]. However, there is no significant difference between highly and lowly expressed CXCL8 in ccRCC patients (Figure 3(c)). Thus, critical differences in survival-associated DEGs of ccRCC patients were explored using TCGA database and ID1 is discovered after filtration (Figures 3(d) and 3(e)). ID1 is one of the most investigated members of the ID family, and recent researches have shown that ID1 is associated with invasion, tumor malignancy, undifferentiation, and poor prognosis in several cancers [26]. In order to define the clinical features of ID1 in ccRCC, TNM stage, lymph nodes, serum calcium, and hemoglobin are screened from data set and compared between low-grade and high-grade patients. It reviewed that lowly expressed ID1 is associated with high serum calcium and high probability of metastasis (Figure 4). These findings are validated by recent studies that lower serum calcium in ccRCC patients is correlated with better prognosis [27, 28]. Besides, patients with tumor metastasis definitely survive shorter than their counterpart. Together, these results uncover a close correlation between ID1 and clinical performances of patients with ccRCC.

To further shed light on the mechanism of ID1 in ccRCC, ID1 top neighbor genes are explored using GeneMANIA database [18]. Part of these genes have common functions: binding to regulatory region nucleic, DNA or transcription regulatory region DNA (Figure 5). For instance, MYF5 and MYOD as transcription factors are mutually-exclusively expressed, and each is required for sustained tumor growth [29]; TCF12 is a poor prognostic factor of ovarian cancer and prostate cancer [30]. Later, K-M plots were performed in the Kaplan-Meier plotter to elucidate the association between these genes and patient's survival status. Based on the survival analysis, we discovered that ID1-associated genes with transcriptional functions, lowly or highly expressed, were almost significant in patient's survival time (Figure 6). Combining with the research that DNA binding activity is associated with metastasis, we speculated that ID1 might influence metastasis through its function of inhibiting DNA binding. In general, binding to regulatory region or transcription regulatory region plays an important role in ID1-regulated ccRCC. Besides, given the outcome that ID1 was related to tumor metastasis and the fact that immune system, especially tumor-infiltrating immune cells, contributes to tumor metastasis cascade [31], we inferred that ID1 might also affect tumor metastasis through immune system. The hypothesis is validated by our analysis in virtue of TIMER database [19], which unveiled that ID1 expression was positively correlated with infiltration level in CD8+ cells and negatively correlated with infiltration level in B cells, macrophages, and neutrophils (Figure 7(a)). In addition, low expression of CD8+ T cells and macrophages associated with poor survival (Figure 7(b)). Together, these suggested that the effect of ID1 on survival of patients was correlated with CD8+ T cells and macrophages. It is reported that macrophages function in tumor metastasis [32] and tumor-associated macrophages (TAMs) can be regarded as a therapy target [33, 34]. Piranlioglu [35] unraveled that metastasis can be induced after suppressing CD8 T cells. Taken together, ID1 affects the deterioration of ccRCC through its DNA binding regulation and immune system.

Considering relatively-high ID1 expression indicates better survival, we selected target drugs by CTD. Among the drugs which can elevate ID1 expression, Valproic acid was the most convinced (Figure 8). Based on Wei's discovery, Valproic acid exhibits cytotoxicity to renal cell carcinoma [36]. We believe Valproic acid is likely to prevent ccRCC progression and metastasis in early stage patients through elevating ID1 expression.

## 5. Conclusion

ID1, a biomarker of both clinical outcome and infiltration in ccRCC, has the potential function of preventing deterioration in ccRCC progression and metastasis. Therefore, ID1 can be a therapy target at early stage to keep a relative high ID1 expression level, which was expected to protect ccRCC patients from exacerbation.

## Figures and Tables

**Table 1:** KEGG pathway enrichment analysis of upregulated DEGs.

ID	Description	Gene ratio	Number of enriched genes	*P* value
hsa04015	Rap1 signaling pathway	0.25000	5	0.00014
hsa04371	Apelin signaling pathway	0.20000	4	0.00033
hsa04010	MAPK signaling pathway	0.25000	5	0.00068
hsa05231	Choline metabolism in cancer	0.15000	3	0.00179
hsa04928	Parathyroid hormone synthesis, secretion and action	0.15000	3	0.00225
hsa05418	Fluid shear stress and atherosclerosis	0.15000	3	0.00484
hsa04151	PI3K-Akt signaling pathway	0.20000	4	0.01074
hsa05031	Amphetamine addiction	0.10000	2	0.01247
hsa03320	PPAR signaling pathway	0.10000	2	0.01542
hsa04014	Ras signaling pathway	0.15000	3	0.01953
hsa05323	Rheumatoid arthritis	0.10000	2	0.02256
hsa04657	IL-17 signaling pathway	0.10000	2	0.02302
hsa04925	Aldosterone synthesis and secretion	0.10000	2	0.02488
hsa04380	Osteoclast differentiation	0.10000	2	0.04067

**Table 2:** KEGG pathway enrichment analysis of downregulated DEGs.

ID	Description	Gene ratio	Number of enriched genes	*P* value
hsa05133	Pertussis	0.15789	3	0.00074
hsa00770	Pantothenate and CoA biosynthesis	0.10526	2	0.00091
hsa05322	Systemic lupus erythematosus	0.15789	3	0.00368
hsa05030	Cocaine addiction	0.10526	2	0.00599
hsa04720	Long-term potentiation	0.10526	2	0.01097
hsa05031	Amphetamine addiction	0.10526	2	0.01129
hsa04610	Complement and coagulation cascades	0.10526	2	0.01503
hsa05132	Salmonella infection	0.10526	2	0.01539
hsa05150	Staphylococcus aureus infection	0.10526	2	0.02171
hsa04061	Viral protein interaction with cytokine	0.10526	2	0.02344
hsa04064	NF-kappa B signaling pathway	0.10526	2	0.02432
hsa04620	Toll-like receptor signaling pathway	0.10526	2	0.02522
hsa04728	Dopaminergic synapse	0.10526	2	0.03859
hsa00360	Phenylalanine metabolism	0.05263	1	0.04002

**Table 3:** Significance of clinical indicators between two groups.

Clinical indicators	*P* value	Significance
T (tumor)	0.414	
N (nodes)	0.73	
M (metastasis)	0.032	^∗^
Lymph nodes	0.068	
Serum calcium	0.021	^∗^
Hemoglobin	0.925	

Note: ^∗^*P* < 0.05.

**Table 4:** Multivariate Cox analysis of immune cells infiltration.

Cell type	Coef	HR	95% CI	*P* value	Sig
B_cell	-0.600	0.549	(0.022, 13.575)	0.714	
CD8_Tcell	-1.741	0.175	(0.037, 0.837)	0.029	^∗^
CD4_Tcell	-0.524	0.592	(0.039, 8.902)	0.705	
Macrophage	-2.774	0.062	(0.006, 0.647)	0.020	^∗^
Neutrophil	3.211	24.809	(0.389, 1582.755)	0.130	
Dendritic	1.119	3.062	(0.517, 18.131)	0.217	

Notes: ∗P < 0.05 was considered to denote statistical significance.

## Data Availability

The data used to support the findings of this study are available from the corresponding author upon request.
